# A190 MICROBIAL AND MOLECULAR PROFILING OF THE APPENDIX OF PEDIATRIC PATIENTS WITH INFLAMMATORY BOWEL DISEASES

**DOI:** 10.1093/jcag/gwae059.190

**Published:** 2025-02-10

**Authors:** N Arjomand Fard, J M Githaka, S Veniamin, L Guan, J Andrews, K Madsen, T Perry, E Wine

**Affiliations:** University of Alberta, Edmonton, AB, Canada; University of Alberta, Edmonton, AB, Canada; University of Alberta, Edmonton, AB, Canada; The University of British Columbia, Vancouver, BC, Canada; University of Alberta, Edmonton, AB, Canada; University of Alberta, Edmonton, AB, Canada; University of Alberta, Edmonton, AB, Canada; University of Alberta, Edmonton, AB, Canada

## Abstract

**Background:**

Inflammatory bowel diseases (IBD), including Crohn disease (CD) and ulcerative colitis (UC), are chronic inflammatory conditions influenced by genetic, environmental, and microbial factors. Emerging evidence suggests a role for the appendix in IBD with immune and microbe impacts on the colon. Peri-appendicular patch (PAP) inflammation is observed in some UC patients; appendectomy has shown protective effects, reducing relapse and colectomy rates in UC. However, the mechanisms underlying these associations remain unclear.

**Aims:**

This study aims to explore these mechanisms by characterizing microbial species, molecular pathways, and host-microbiota interactions in the appendix of IBD patients.

**Methods:**

Mucus from the appendix and non-inflamed colon of 10 pediatric IBD and 5 non-IBD patients undergoing elective surgical resection was collected for DNA and whole-genome sequencing. RNA and DNA from appendix tissues were used for RNA-seq and 16S rRNA sequencing, respectively. Metagenomic analysis was performed across all platforms. Fluorescence *in-situ* hybridization (FISH) and immunofluorescence defined localization.

**Results:**

Proteobacteria were significantly enriched in IBD (mean = 27.23% ± 32%) compared to non-IBD mucus samples (mean = 1.6% ± 0.23%, *p* = 0.0052), with reduced bacterial diversity in the appendix of IBD patients. Differential abundance analysis revealed site-specific changes in MetaCyc pathways, notably in the IBD-appendix; for example, ABH and Lewis epitopes biosynthesis pathways were highly enriched in this organ. 38 of 50 MetaCyc pathways were linked to specific species, with *E. coli* contributing to 32 pathways. Immune and inflammatory pathways were significantly upregulated in the IBD-appendix. Strong correlations were observed between host tissue and microbial pathways in IBD mucus; for instance, Chondroitin Sulfate and Dermatan Sulfate Biosynthesis bacterial pathways were positively correlated with the upregulated host pathway Chemokine Receptors Bind Chemokines (Rho = 0.8851 and 0.88549, respectively), reflecting host-bacteria interaction in the appendix. Bacteria were observed in the mucus layer of IBD-appendix.

**Conclusions:**

The appendix in IBD exhibits unique molecular and microbial features, offering key insights into its role in IBD pathogenesis and potential therapeutic avenues, reinforcing the efficacy of interventions such as appendectomy in UC.

**Funding Agencies:**

CIHRWCHRI

